# Desert Milkweed ( *Asclepias erosa* ) as a Midsummer Resource for Arthropod Communities in the Great Basin Desert

**DOI:** 10.1002/ece3.74471

**Published:** 2026-10-11

**Authors:** Aramee C. Diethelm, Haylie R. Wilcox, Herron N. Renegado

**Affiliations:** ^1^ Department of Evolution and Ecology University of California Davis California USA; ^2^ Department of Entomology and Nematology University of California Davis California USA

**Keywords:** community assembly, plant–arthropod interactions, seasonal refugia, structural architecture, trophic interactions, xeric environments

## Abstract

In arid ecosystems, consumer communities are often structured by strong temporal fluctuations in resource availability. During midsummer in the Great Basin Desert, high temperatures and declining soil moisture cause many early‐season plants to senesce, potentially concentrating arthropod activity on the few species that remain physiologically active. We characterized arthropod assemblages associated with the desert milkweed ( *Asclepias erosa* ) to evaluate its potential role as a focal plant during this seasonal period of reduced floral and vegetative productivity. Arthropods were surveyed on randomly selected plants across four sites in northern Nevada during June 2025 using standardized observations, during which arthropod identity, behavior, and location on the plant were recorded. Across 41 plants, we recorded 1779 arthropod individuals representing 67 morphospecies across six orders. Arthropods occupied nearly all plants surveyed and used all aboveground plant structures, including flowers, leaves, and stems. Community composition was highly skewed toward a few dominant taxa, with two dipteran morphospecies accounting for over 40% of all recorded individuals. Furthermore, the majority of morphospecies were rare in their distribution, occurring on fewer than three plants. Flowering plants supported significantly greater arthropod abundance and morphospecies richness than vegetative plants; however, most vegetative individuals also harbored arthropods. While nectar‐feeding individuals accounted for many observations, numerous arthropods were resting, moving among plant structures, or occupying stems and leaves without actively feeding, indicating that *A. erosa* may also provide structural habitat, refuge from thermal stress, and opportunities for predation or other trophic interactions. These results provide baseline natural history data on plant–arthropod interactions in Great Basin Desert system and suggest that *A. erosa* may function as a localized resource patch supporting diverse arthropod assemblages during the midsummer period of resource limitation.

## Graphical abstract

BLURB FOR ETOC: In the Great Basin Desert, many plants senesce as heat and drought intensify in midsummer, potentially concentrating arthropods on the few species that remain physiologically active. We surveyed arthropods on desert milkweed ( *Asclepias erosa* ) during this period and recorded 1779 individuals representing 67 morphospecies across 41 plants; floral tissues supported the highest arthropod abundance, while flowering plants supported greater morphospecies richness and incidence than non‐flowering plants, and vegetative plants were consistently occupied even without evidence of feeding. These patterns suggest that *A. erosa* functions as a focal resource patch and may also provide structurally mediated refugia from thermal stress during a period of pronounced resource limitation.

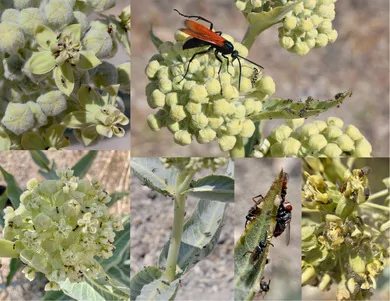

## 1 Introduction

Xeric environments, including deserts and arid shrublands, are characterized by chronic water limitation, high temperature variability, and irregular resource availability (Noy‐Meir 1973; Whitford and Duval 2019). These constraints limit plant productivity and generate landscapes where trophic resources and suitable habitat are often sparse and temporally variable (Hadley and Szarek 1981; Whitford and Duval 2019). Despite these constraints, desert ecosystems support diverse assemblages of plants and animals that persist by exploiting brief periods of productivity or localized patches of resource availability (Hadley and Szarek 1981; Ward 2016). Arthropods depend on plant‐derived resources for nutrition, habitat, and refuge from predators (Polis and Strong 1996; Tews et al. 2004). Because of this dependence, local arthropod distributions and community composition are highly sensitive to seasonal shifts in plant phenology and the availability of vegetative and floral structures (Crawford and Gosz 1982).

In the arid regions of western North America, resource limitation intensifies during midsummer as rising temperatures and declining soil moisture cause many early‐season plants to senesce (Beatley 1974; Comstock et al. 1988; Donovan and Ehleringer 1994). During this period, arthropod activity may become concentrated on plants that remain physiologically active or provide structural habitat (Hadley and Szarek 1981; Tews et al. 2004; Ward 2016). Plant species that retain flowers or vegetative structures during periods of resource limitation may provide localized resource patches and refugia for arthropods when comparable resources are scarce in the surrounding landscape (Lawton 1983; Lortie et al. 2016; Whitford and Duval 2019). These spatially concentrated resource patches can support arthropod communities that contribute to pollination services, nutrient turnover, and prey availability for higher trophic levels (Hadley and Szarek 1981; Ward 2008; Whitford 2000).

Plant–arthropod associations in arid systems are mediated by multiple plant traits and structures (Randlkofer et al. 2010; Tews et al. 2004). Flowers can provide nectar and pollen resources used by a wide range of taxa (Herrera 1989; Waser and Ollerton 2006), whereas vegetative tissues may support herbivores (Crawford and Gosz 1982) and are often more constrained by species‐specific host associations and plant defenses (Crawford and Gosz 1982; Schoonhoven et al. 2005). Beyond nutrition, plant architecture provides structural habitat, such as perching sites and shelter, and creates localized microhabitats that can affect arthropod presence, behavior, and vulnerability to predators (Ayal 2007; Lawton 1983; Price et al. 1980). Vertically structured plants may also elevate arthropods above the soil, reducing exposure to extreme surface temperatures (Kidron 2009). Together, these traits enable individual plants to support arthropods across multiple trophic levels during periods of seasonal stress (Birkhofer et al. 2008; Braun et al. 2021; Grevstad and Klepetka 1992). Documenting arthropod associations during resource‐limited periods is therefore necessary to quantify how individual desert plants support arthropod abundance and diversity, and how specific plant structures mediate community assembly. Yet detailed inventories of arthropods associated with desert plant species remain limited, constraining our ability to evaluate these plant–arthropod associations (Durant et al. 2012; Esque et al. 2021).

The desert milkweed ( *Asclepias erosa* Torr.) is native to the southwestern United States and occurs across the Mojave and Great Basin deserts (Beckett et al. 1938; Woodson 1954). This species exhibits several traits associated with drought tolerance, including thick leaves, dense trichomes, and a deep taproot (Agrawal et al. 2009; Hensen and Wolf 2023; Nabhan et al. 2015; Woodson 1954), allowing it to maintain vegetative growth and floral production into midsummer (Sundell 1994), when few alternative nectar resources may be available (Shreve 1942; Wright et al. 2015). Its clustered flowers and clumping stems bearing broad leaves may generate localized microclimate buffering under high summer temperatures and offer structural refuge for arthropods (Agrawal et al. 2009; Beckett et al. 1938; Woodson 1954), potentially reducing exposure to extreme heat and predators (Kidron 2009; Liu et al. 2016). Like other *Asclepias* species, *A. erosa* possesses toxic chemical and physical defenses including cardiac glycosides called cardenolides and latex (Malcolm 1991), which may limit direct consumption of vegetative tissues to herbivores with physiological adaptations for overcoming these defenses. Although the direct act of consuming the plant may be strongly constrained, other forms of plant use including nectaring, other trophic interactions (e.g., secondary predation), and refuge, may include taxa without the same physiological adaptations for consuming milkweed tissues. When flowering, *A. erosa* produces accessible nectar and prominent inflorescences known to attract a diverse assemblage of pollinators, including native bees across multiple genera (Esque et al. 2021; Wilson 2021), as well as butterflies such as hairstreaks (Theclinae), and additional taxa such as spider and tarantula hawk wasps (Pepsis), milkweed bugs (Lygaeidae), and spiders (Araneae) (Isman et al. 1977; Nabhan et al. 2015).

Here, we present a multi‐site inventory of arthropods associated with *A. erosa* during midsummer, a period of pronounced resource limitation in the Great Basin Desert (Comstock and Ehleringer 1992; Shreve 1942). Preliminary field observations in northern Nevada indicated that *A. erosa* supported unusually high arthropod activity, even when few other plants in the surrounding landscape harbored insects (pers. obs.). Motivated by these observations, we quantified arthropod abundance, morphospecies richness, taxonomic composition, and observed behaviors on both flowering and non‐flowering (vegetative) *A. erosa* plants. We also documented arthropod use of different *A. erosa* plant structures. In particular, we examined arthropod abundance across floral and vegetative tissues to evaluate whether plant structural architecture supports arthropod use independent of nectar resources. It remains poorly understood in desert systems how arthropods use different plant structures and whether use changes with flowering status (Franklin et al. 2016; Lortie et al. 2016). Our objective was to characterize the composition and distribution of arthropods associated with *A. erosa* to evaluate its role as both a focal resource patch and a seasonal refugium for diverse taxa, thereby providing foundational natural history information on plant–arthropod interactions during a period of acute resource limitation in the Great Basin Desert.

## 2 Material and Methods

### 2.1 Study System

*Asclepias erosa* is a perennial forb native to the southwestern United States, occurring in desert washes (ephemeral drainage channels), sandy or rocky well‐drained soils from the southern Central Valley and southern California through southern Nevada and southwestern Utah, extending south to western Arizona, Baja California, and northern Sonora (Beckett et al. 1938; Woodson 1954). Individual *A. erosa* plants typically grow 1–2 m tall and produce 8–20 upright stems arising from a central base (Beckett et al. 1938; Sundell 1994). Leaves are large for a desert forb, with mature leaves spanning 15–25 cm long and 9–11 cm wide (Beckett et al. 1938; Sundell 1994). Leaves and stems frequently bear many fine trichomes (Agrawal et al. 2009; Borders et al. 2012). Individuals produce dense clusters of flowers that typically emerge from late spring through summer and occasionally continue into autumn (Sundell 1994; Woodson 1954). The species grows in full sun, tolerates high temperatures, and survives with minimal water once established (Borders et al. 2012; Sundell 1994). *Asclepias erosa* individuals generally occur in discrete patches composed of scattered groupings of 10 or more plants (Beckett et al. 1938).

We conducted this study at four naturally occurring patches of *A. erosa* in the Great Basin Desert of northern Nevada with two sites in Dixie Valley and two outside of the town of Gabbs (Figure 1). In the Dixie Valley area, Site A was located within a desert wash (39.442120°, −118.158233°), while Site B was on a mountain slope (39.374190°, −118.064222°). Outside of Gabbs, Site C (38.78472°, −117.96181°) and Site D (38.784775°, −117.96183°) were located along the same dirt road, with Site D farther down the road within a desert wash. Each site had at least 20 different individual *A. erosa* plants. We conducted field observations under daytime summer conditions. During surveys, ambient air temperatures ranged from 28.3°C to 30.6°C, with wind speeds from 2.4 to 7.4 km/h. To account for temporal variation in arthropod activity, we conducted all surveys between 12:00 and 17:00 h local time.

**FIGURE 1:**
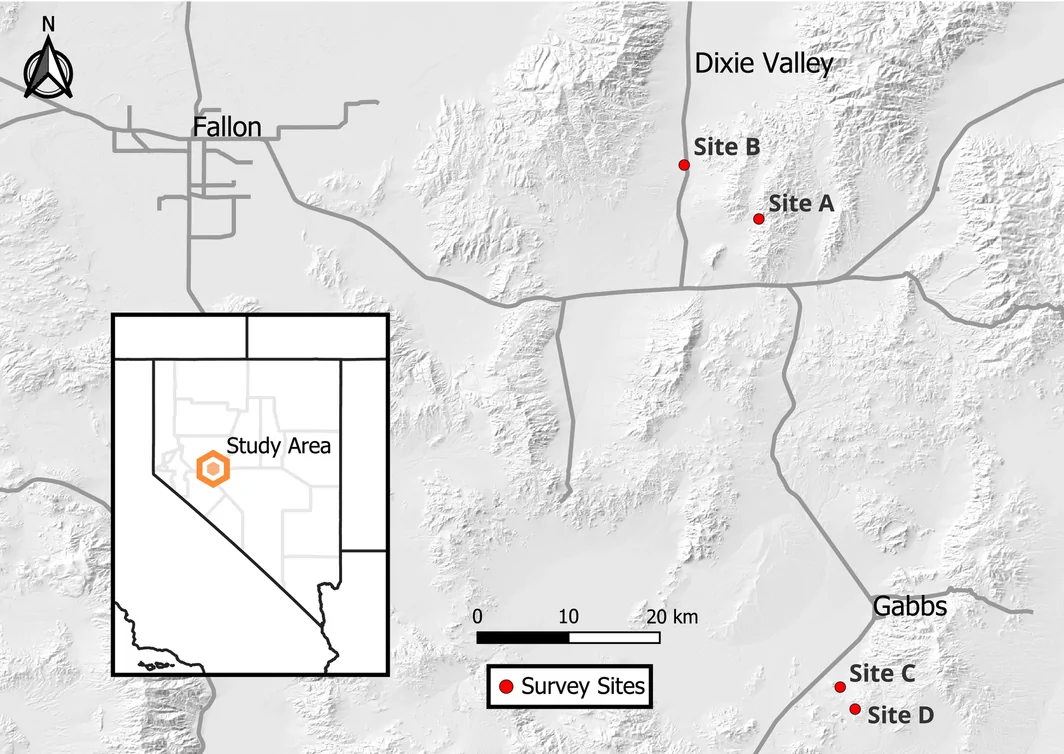
The study area (indicated by orange hexagon) and sampling sites (indicated by red dots) in Nevada, USA. The plotted locations of Sites C and D were jittered for visibility; the sites are closer together than they appear.

The study region experiences a cold desert climate characteristic of the central Great Basin. Long‐term average annual precipitation is low, with < 300 mm/year in Dixie Valley and < 175 mm/year in the Gabbs area (Eakin 1962; Lawrence and Harrill 1995; USDA 2018). Summer conditions are characterized by high temperatures and low soil moisture, with average daily high temperatures in the region reaching 33°C in July and an all‐time record maximum temperature of 42°C (Western Regional Climate Center 2013), and potential evapotranspiration exceeding precipitation throughout much of the dry period (Cohen and Everett 1963; Eakin 1962). All study sites occurred within Great Basin Desert scrub habitat, characterized by sparse vegetation and low overall plant cover (Caldwell 1985). Dominant surrounding vegetation at study areas included widely spaced shrubs such as *Artemisia tridentata* , *Sarcobatus vermiculatus* , *Ephedra nevadensis* , and *Ericameria* spp. During the sampling period, few other plant species were flowering in the immediate vicinity of *A. erosa* patches, and alternative floral nectar resources were scarce or absent in the surrounding landscape.

### 2.2 Data Collection

The characterization of the midsummer arthropod community was conducted on 24–25 June 2025 at four study sites in northern Nevada. At each site, we selected individual *A. erosa* plants from within each patch and aimed to survey equal numbers of flowering plants, defined as bearing open flowers or accessible nectar, and vegetative plants, defined as lacking open flowers or accessible nectar. *Asclepias erosa* individuals were chosen randomly, with a minimum spacing of 5 m between consecutively surveyed individuals to minimize disturbances to arthropods and to sample broadly across the spatial extent of the population. Field constraints limited flowering plants to 41% of the sampled individuals.

We surveyed between 10 and 11 *A. erosa* individuals per site (Sites A, B, D: *n* = 10; Site C: *n* = 11), for a total of 41 focal individuals. For each, we conducted two‐minute visual surveys, during which we recorded all arthropods present along with their behavior and position on the plant. We categorized behaviors into functional classifications, including nectaring, herbivory or plant material collection (e.g., carrying plant matter), stationary (e.g., resting or sitting), active movement (e.g., walking or climbing), and other less observed activities such as mating.

To minimize disturbance‐induced behavioral changes and provide an unbiased baseline of arthropod activity, we conducted visual surveys prior to any physical collection. Following these observations, representative arthropods were captured and preserved as voucher specimens to facilitate taxonomic verification. We assigned all observed individuals to taxonomic order and subsequently delineated morphospecies, a standard proxy for species‐level diversity in arthropod surveys, based on morphological characteristics (Oliver and Beattie 1993), using taxonomic keys for verification when possible (Triplehorn et al. 2021). We excluded one unidentified taxon from final analyses. To supplement identifications, we uploaded specimen photographs to iNaturalist (n.d.) for further verification (http://www.inaturalist.org/observations; accessed 30 March 2026). All physical specimens and digitized images are maintained as a reference collection at the University of California, Davis.

### 2.3 Statistical Analysis

All analyses were conducted in R version 4.5.2 (R Core Team 2025).

Arthropod abundance was defined as the number of individual arthropods observed, morphospecies richness as the number of distinct morphospecies observed, and incidence as the presence or absence of a morphospecies on an individual *A. erosa* plant. To evaluate sampling completeness at the site level, we used an incidence‐based approach treating each *A. erosa* individual as a sampling unit and calculating the presence–absence of each morphospecies with the *iNEXT* package (Hsieh et al. 2025). To visualize patterns of morphospecies accumulation across survey sites, we generated incidence‐based rarefaction and extrapolation curves for Shannon diversity (Hill number *q* = 1, representing the effective number of species) at the site level. These curves were constructed using presence–absence data pooled across all sampled *A. erosa* plants within each site and implemented using the iNEXT package (Chao et al. 2014; Hsieh et al. 2025).

To analyze differences in arthropod abundance across *A. erosa* plant tissue types, we employed a generalized linear mixed model (GLMM) with a Poisson distribution using the *glmmTMB* package (Brooks et al. 2017). We compared three categories: (1) floral tissues on flowering plants, (2) vegetative tissue on flowering plants, and (3) vegetative tissue on non‐flowering (vegetative) plants. We included site as a fixed effect and a unique plant identification code as a random effect to account for the non‐independence of repeated measures (floral vs. vegetative tissue) on flowering individuals. Model fit was validated using the *DHARMa* package (Hartig 2017). Post hoc pairwise comparisons were conducted using the *emmeans* package with Tukey's HSD adjustment (Lenth 2025).

To evaluate whether morphospecies richness differed between flowering and non‐flowering *A. erosa* individuals while accounting for site‐level variation, we utilized a generalized linear model (GLM) with a Poisson distribution with plant flowering status and site as fixed effects. Model fit was again validated using the *DHARMa* package (Hartig 2017). Additionally, we used a binomial generalized linear mixed model (GLMM) with a logit link to test whether morphospecies incidence differed between flowering and non‐flowering *A. erosa* individuals. Morphospecies presence or absence on each sampled *A*. *erosa* plant was modeled as the response, with plant flowering status and site as fixed effects and plant identity and morphospecies identity as crossed random intercepts. Model fit was assessed as described previously.

## 3 Results

Across four northern Nevada study sites, we observed a total of 1779 arthropod individuals representing 67 unique morphospecies across six orders (Araneae, Coleoptera, Diptera, Hemiptera, Hymenoptera, and Lepidoptera) on 41 *A. erosa* individuals during midsummer. Eleven of these morphospecies were further identified to genus (Table 1). A small number of morphospecies accounted for a large proportion of observed individuals, while most morphospecies occurred on relatively few sampled plants. Two dipteran morphospecies (Diptera G: *n* = 404; Diptera H: *n* = 318) accounted for 40.6% of all individuals observed. In contrast, 39 morphospecies (58%) were observed on fewer than three sampled *A. erosa* individuals, whereas 13 morphospecies (19%) were observed on more than 10 plants (Table 1). However, arthropods were present on nearly all *A. erosa* individuals (*n* = 38) and utilized the full suite of aboveground structures, including flowers, leaves, and stems (Figure 2). Site‐level abundance ranged from 139 to 595 individuals, while morphospecies richness per site ranged from 11 to 42. The proportion of flowering *A. erosa* also varied by site, from 20% (Site B) to 54.5% (Site C; Figure 3a). Arthropods were present on all flowering individuals and on 87.5% of non‐flowering individuals, showing a high degree of arthropod presence even in the absence of floral nectar resources. Incidence‐based sample coverage indicated moderate to high sampling completeness with sample coverage ranging from 0.54 at Site B up to 0.93 at Site D (Sites A and C: 0.84 and 0.87, respectively). The incidence‐based rarefaction and extrapolation curves for Shannon diversity (Hill number *q* = 1) reached an asymptote at three of the four sites, consistent with sufficient sampling effort to characterize the dominant portion of the community (Figure 3b).

**TABLE 1:** Taxonomic composition and classification of arthropod morphospecies observed on *Asclepias erosa* in the Great Basin Desert (*N* = 41 plants) on 24–25 June of 2025.

Order	Family	Genus	Morphospecies (plant incidence, %)
Araneae	Salticidae	*Phiddipus*	Araneae C (2.4), Araneae D (2.4)
	Thomisidae	*Mecaphesa*	Araneae A (29.3), Araneae B (4.9)
Coleoptera	Melyridae	*Collops*	Coleoptera C (7.3)
	Melyridae	Unknown	Coleoptera B (46.3)
	Ripiphoridae	*Macrosiagon*	Coleoptera F (2.4)
	Tenebrionidae	Unknown	Coleoptera A (4.9)
Coleoptera	Unknown	Unknown	Coleoptera D (7.3), Coleoptera E (39.0), Coleoptera G (4.9)
Diptera	Sepsidae	Unknown	Diptera C (2.4)
	Syrphidae	Unknown	Diptera A (2.4)
	Tachinidae	Unknown	Diptera B (22.0)
	Unknown	Unknown	Diptera D (29.3), Diptera E (2.4), Diptera F (2.4), Diptera G (36.6), Diptera H (26.8), Diptera I (2.4), Diptera J (2.4), Diptera K (2.4), Diptera L (2.4), Diptera M (2.4), Diptera N (2.4), Diptera O (2.4), Diptera P (2.4), Diptera Q (7.3), Diptera R (2.4)
Hemiptera	Lygaeidae	Unknown	Hemiptera A (7.3)
Hymenoptera	Bembicidae	*Microbembex*	Hymenoptera M (2.4)
	Bembicidae	Unknown	Hymenoptera AA (41.5)
	Braconidae	Unknown	Hymenoptera F (36.6), Hymenoptera J (2.4)
	Chalcididae	Unknown	Hymenoptera N (9.8)
	Chrysididae	Unknown	Hymenoptera AE (4.9), Hymenoptera AF (2.4)
	Colletidae	*Hylaeus*	Hymenoptera AC (7.3)
	Crabronidae	Unknown	Hymenoptera I (2.4), Hymenoptera R (7.3)
	Halictidae	Unknown	Hymenoptera AB (2.4), Hymenoptera AD (4.9)
	Formicidae	*Crematogaster*	Hymenoptera A (22.0), Hymenoptera B (29.3)
	Formicidae	Unknown	Hymenoptera C (36.6), Hymenoptera D (41.5), Hymenoptera E (2.4)
	Mutillidae	*Dasymutilla*	Hymenoptera K (4.9)
	Pompilidae	*Pepsis*	Hymenoptera G (24.4)
	Pompilidae	*Eurytoma*	Hymenoptera L (17.1)
	Scoliidae	*Colpa*	Hymenoptera P (2.4)
	Scoliidae	Unknown	Hymenoptera O (2.4)
	Sphecidae	Unknown	Hymenoptera H (26.8)
	Unknown	Unknown	Hymenoptera Q (2.4), Hymenoptera S (26.8), Hymenoptera T (7.3), Hymenoptera U (2.4), Hymenoptera V (2.4), Hymenoptera W (2.4), Hymenoptera X (2.4), Hymenoptera Y (2.4), Hymenoptera Z (7.3), Hymenoptera AG (2.4), Hymenoptera AH (7.3), Hymenoptera AI (2.4)
Lepidoptera	Euchaetes	Unknown	Lepidoptera B (4.9)
	Unknown	Unknown	Lepidoptera A (17.1)

*Note:* Plant incidence was calculated for each morphospecies as the number of sampled *A. erosa* individuals on which it was observed divided by the total number of plants sampled. Morphospecies were delineated based on unique morphological characteristics and assigned to the lowest possible taxonomic level using visual characteristics and voucher specimens. “Unknown” indicates taxa that could not be reliably assigned beyond the specified taxonomic rank.

**FIGURE 2:**
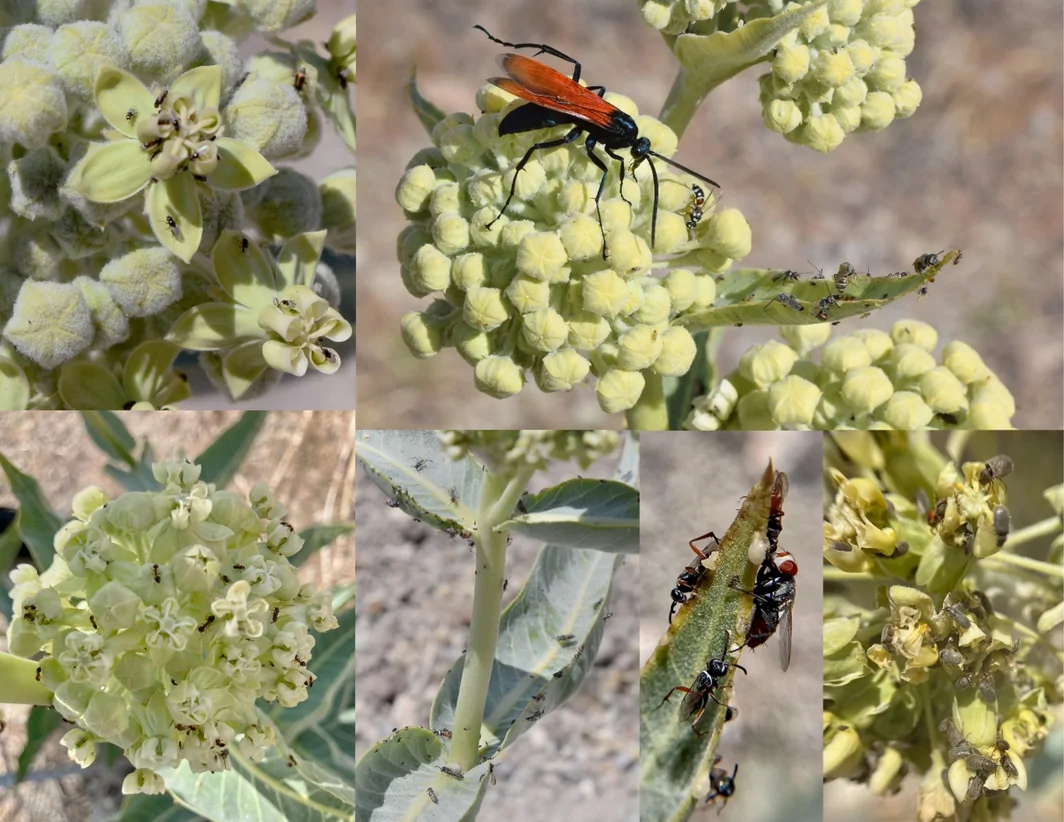
Representative arthropod diversity and behaviors on *Asclepias erosa* structures. Top row (L to R): Open umbels dominated by Diptera (morphospecies: “Diptera G”; nectaring) and Coleoptera (“Coleoptera B”); varied Hymenoptera and Diptera morphospecies along the margins of a leaf near a budding umbel Hymenoptera visitors. Bottom row (L to R): Open umbels dominated by *Crematogaster* ants (“Hymenoptera B”); high occupancy of the vertical stem and leaf margins by varied morphospecies; Hymenoptera (“Hymenoptera L”) and Diptera (“Diptera G”) at leaf margins with accumulations of dried latex; and open umbels dominated by Coleoptera (“Coleoptera E”).

**FIGURE 3:**
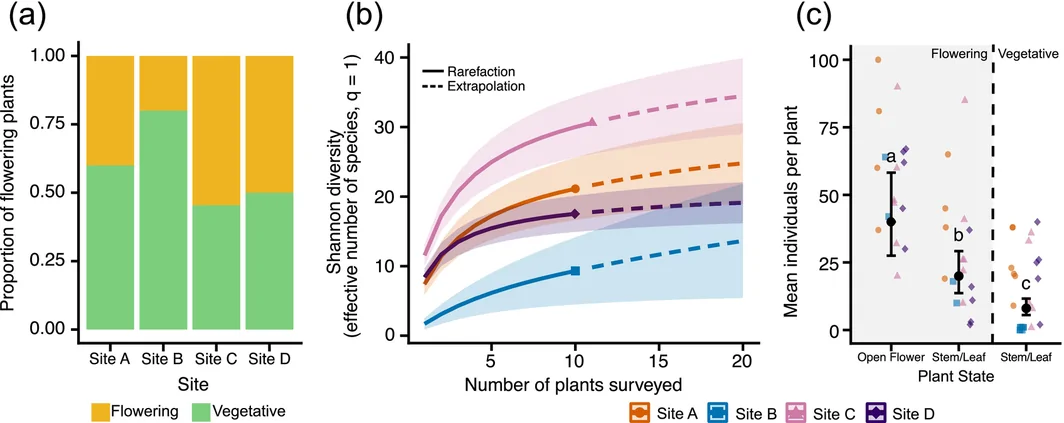
(a) Proportion of vegetative and flowering *Asclepias erosa* individuals sampled across the four study sites. (b) Incidence‐based rarefaction (solid lines) and extrapolation (dashed lines) curves for Shannon diversity (Hill number *q* = 1), representing the effective number of morphospecies. Shaded areas indicate 95% confidence intervals. (c) Model‐estimated mean number of arthropods per plant across flowering (gray region) and non‐flowering plants (white region), separated by plant structures (floral tissue or stem and leaf tissue). Error bars represent 95% confidence intervals; different letters above bars indicate statistically significant differences at *p* < 0.05; small, jittered points represent the raw individual plant observations.

Arthropod abundance differed among *A. erosa* plant tissue and flowering‐status categories (Wald *χ* ^2^ = 180.87, df = 2, *p* < 0.0001; Figure 3c). Model‐estimated mean number of arthropods per *A. erosa* individual, averaged across sites and back‐transformed from the log scale, was highest on floral tissue of flowering plants (40.1 individuals; 95% CI: 27.5–58.3), intermediate on vegetative tissues of flowering plants (20.0 individuals; CI: 13.7–29.2), and lowest on vegetative tissues of non‐flowering plants (8.0 individuals; CI: 5.5–11.6). Tukey‐adjusted pairwise comparisons indicated that arthropod abundance was significantly higher on floral tissues than on vegetative tissues of flowering *A. erosa* plants (log‐scale estimate = 0.70 ± 0.06 SE, *z* = 12.38, *p* < 0.0001) and than on vegetative tissues of non‐flowering plants (log‐scale estimate = 1.61 ± 0.26, *z* = 6.11, *p* < 0.0001). Notably, arthropod abundance was also significantly higher on vegetative tissues of flowering plants than on vegetative tissues of non‐flowering plants (log‐scale estimate = 0.91 ± 0.27, *z* = 3.44, *p* = 0.002).

Behavioral observations were dominated by movement (e.g., walking; 43.5%) and nectaring (49.6%), while stationary behaviors were rare (5.0%; Table 2). All other behaviors, including non‐nectar‐based foraging or mating, accounted for < 2% of observations (Table 2). Patterns of behavior varied among orders (Table 2). Diptera and Coleoptera were observed primarily nectaring, Hymenoptera were mostly active but also engaged in nectaring and stationary behaviors, Lepidoptera were mainly foraging, and Araneae were largely stationary or moving across plant structures. Hemiptera were represented by only three individuals (one active, two stationary).

**TABLE 2:** Summary of arthropod assemblages observed on *Asclepias erosa* across four study sites in northern Nevada during June 24–25 of 2025, where active behaviors indicate movement such as walking and foraging for non‐nectar‐based feeding or carrying of plant materials (see Section 2 for details).

Order	Number of morphospecies	Number of individuals	Number of individuals per observed behaviors				
			Active	Foraging	Mating	Nectar‐ing	Stationary
Araneae	4	23	7	0	0	0	16
Coleoptera	7	268	120	0	0	138	10
Diptera	18	772	169	0	2	586	15
Hemiptera	1	3	1	0	0	0	2
Hymenoptera	35	673	475	1	2	152	43
Lepidoptera	2	40	1	30	0	6	3

Among flowering *A. erosa* individuals, the arthropod community utilized the entire plant, spanning floral, leaf, and stem tissues (Figures 2 and 4). Floral tissues hosted the highest arthropod abundance (Figure 3c) and the broadest taxonomic representation. In terms of morphospecies richness, flowering plants supported significantly more morphospecies per plant (model‐estimated mean ± SE: 9.27 ± 0.83) than non‐flowering plants (3.65 ± 0.40; *β*_vegetative_ = −0.93 ± 0.13, *z* = −7.34, *p* < 0.0001). Flowering plants had about 2.54 times the expected richness of non‐flowering (vegetative) plants. Consistent with this pattern, the model‐estimated probability that a given morphospecies occurred on a sampled plant was significantly higher on flowering *A. erosa* individuals (9.2%) than on vegetative individuals (2.7%; logit‐scale contrast = 1.30 ± 0.15 SE, *z* = 8.68, *p* < 0.0001). Across the arthropod community, Diptera accounted for the greatest number of arthropod individuals, but comprised only 12 of the 67 total morphospecies, whereas non‐ant Hymenoptera (superfamily Apoidea), despite lower abundance, represented 20 morphospecies. Although fewer individuals were observed on leaf tissue of flowering *A. erosa* plants, multiple orders were present, particularly Hymenoptera, primarily Apoidea with smaller contributions from the family Formicidae, along with Diptera, Coleoptera, Araneae, and Lepidoptera. Stem tissue had the lowest number of individuals present, yet those arthropods represented a range of orders, including Hymenoptera, Diptera, Coleoptera, Araneae, and Hemiptera.

**FIGURE 4:**
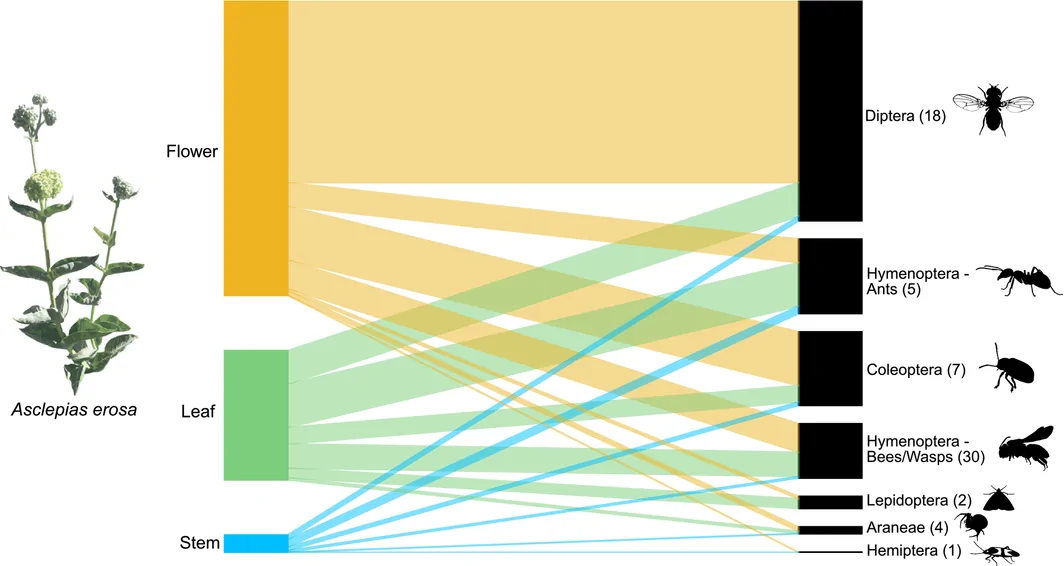
Bipartite interaction network between aboveground structures of flowering *Asclepias erosa* and associated arthropod groups, where line thickness represents the number of arthropod individuals observed on each plant structure (flower, leaf, or stem), and bar height represents the total abundance within each taxonomic group (primarily orders). Hymenoptera is partitioned into ants (Formicidae) and bees/wasps (Apoidea) to highlight distinct patterns of plant utilization. Numbers in parentheses indicate the morphospecies richness within each group.

## 4 Discussion

The high arthropod abundance (1779 individuals) and morphospecies richness (67 unique taxa) observed on only 41 *A. erosa* plants support the role of this milkweed species as a focal resource patch and key structural habitat during a period of pronounced resource limitation in the Great Basin Desert (Cook and Irwin 1992; Lawton 1983; Shreve 1942). Our results show that arthropod activity occurred across all investigated aboveground structures (flowers, leaves, and stems), indicating that arthropods utilize the entire physical architecture of the plant rather than just specific tissues. The presence of arthropods on nearly 90% of non‐flowering plants further suggests that *A. erosa* mprovides structural or thermal refugium even in the absence of nectar rewards. Although floral tissues with nectar resources concentrated activity, supporting the highest morphospecies richness and individual abundance, the consistent occupancy of leaf and stem tissues across plants demonstrates that the functional importance of *A. erosa* extends beyond nectar provision alone. *Asclepias* species are well known for their chemical and physical defenses (Agrawal and Fishbein 2006), including toxic latex and cardenolides (Malcolm 1991), yet the high and consistent arthropod occupancy observed here indicates that these defenses do not preclude widespread use of *A. erosa* as a structural and, in some cases, trophic resource.

The repeated occurrence of a taxonomically broad set of arthropods on non‐flowering tissues suggests that the structural architecture of *A. erosa* provides seasonal refugia during midsummer within this xeric system (Lortie et al. 2016). Few behavioral observations on vegetative tissues involved consumption of *A. erosa* , and only one morphospecies belonged to a known milkweed specialist genus. Instead, many individual arthropods remained stationary or active without feeding. The use of the vegetative tissues by a broader arthropod assemblage is notable and highlights the importance of *A. erosa* beyond its role as a host plant for specialized herbivores, including its potential to provide perching sites, shelter, or refuge from abiotic stress (Gardner et al. 1995; Lawton 1983). These patterns are consistent with structural mediation of habitat use independent of nectar rewards (Price et al. 1980; Tews et al. 2004). In open desert habitats where soil surface temperatures can be extreme, vertically structured plants generate localized microclimatic heterogeneity by providing shade and elevated perching sites that distance arthropods from the heat‐radiating ground (Kidron 2009; Lortie et al. 2016). The vertical stature of *A. erosa* , combined with its broad leaves and dense trichomes, likely facilitates abiotic buffering effects. Similar structural effects have been shown to increase arthropod abundance and richness in arid shrubs (Braun et al. 2021; Liu et al. 2016; Liu et al. 2015). Our results demonstrate a comparable pattern in a perennial forb, highlighting that non‐shrub species may also provide thermally moderated microhabitats during periods of seasonal stress in xeric environments (Lortie et al. 2016).

In addition to potential thermal buffering from plant architecture, leaf structural traits of *A. erosa* , such as trichomes (Li et al. 2023), may facilitate access to scarce atmospheric moisture in desert systems (Jacobs et al. 1999; Kidron et al. 2026; Kidron and Starinsky 2019). We repeatedly observed arthropods positioned along leaf margins of *A. erosa* , sometimes with mouthparts contacting the leaf edge (Figure 2), consistent with documented dew‐harvesting behaviors in other desert taxa (Broza 1979; Moffett 1985). Direct measurements of condensation dynamics and explicit behavioral confirmation would be required to evaluate this mechanism rigorously. In the absence of such measurements, these observations suggest a plausible but untested non‐trophic pathway by which *A. erosa* may attract and retain arthropods. Similarly, the repeated observations of Hymenoptera at sites of dried latex suggests a possible role for latex as a resource. The functional significance of this behavior remains unknown in this system; however, bees in other systems are known to harvest plant latex and related exudates (Popova et al. 2021; Santiago‐Rosario et al. 2025). Still, it remains to be determined whether Hymenoptera occupancy on leaf margins in this system relates to active latex collection, a synergistic effect between the physical properties of dried latex and leaf trichomes, or other unidentified structural benefits.

Although vegetative structures likely provided baseline refugia, flowering status strongly amplified arthropod aggregation and altered community composition. Plants with open flowers supported substantially higher arthropod abundance and morphospecies richness than plants lacking nectar resources. This observed pattern is consistent with resource concentration and resource tracking theory (Abrahms et al. 2021; Root 1973), where consumers disproportionately aggregate in patches with high resource availability. Nearly half of all observed behaviors involved nectaring, suggesting that while the plant provides broad structural benefits, floral resources remain the primary driver of high‐density arthropod aggregations. Attraction to these nectar‐rich patches likely increases residence time and movement within plants, leading to elevated use of adjacent structures such as leaves and stems (Gilpin et al. 2019; Laverty 1992; Thomson 1988). Although this ’magnet’ effect is typically described among neighboring plants, our results suggest a similar process operating within individual plants, where floral rewards concentrate arthropods that subsequently utilize surrounding vegetative tissues. Consistent with this idea, the vegetative tissues of flowering *A. erosa* plants hosted nearly double the model‐estimated arthropod abundance of their non‐flowering counterparts. Together, these findings indicate that floral resources not only increase overall arthropod density but also redistribute activity across the entire plant.

The aggregation of nectar‐feeding taxa on floral tissues likely increases local prey density, indirectly attracting higher trophic orders such as Araneae (Chew 1961). Across four morphospecies and 23 individuals, most spiders (70%) were stationary, consistent with sit‐and‐wait predation, while the remainder were active but not observed foraging or mating. Spiders occurred on flowers, leaves, and stems, indicating they likely exploit arthropod movement across all plant structures rather than focusing solely on floral visitors. Additionally, the dense floral clusters of *A. erosa* could provide structural concealment for ambush predators such as Thomisidae spiders. By supporting arthropods across multiple trophic levels, from nectar‐feeders and those seeking thermal refuge to their associated predators, *A. erosa* functions as a multi‐trophic nexus within a landscape where resources are patchy and abiotic stress can be pronounced (Hadley and Szarek 1981).

Arthropod community structure on *A. erosa* in this study showed strong dominance patterns that are broadly consistent with desert arthropod assemblages but differed in taxonomic composition from many previous studies. Two dipteran morphospecies accounted for over 40% of all individuals, a pattern consistent with desert arthropod communities, which often exhibit dominance by a few taxa alongside many infrequent species (Cheli et al. 2010; Niu et al. 2020). However, the taxonomic composition we observed contrasts with many desert studies reporting dominance by ground‐associated taxa such as Formicidae, Coleoptera, and Araneae (e.g., Braun et al. 2021; Crawford 1988; Gardner et al. 1995; Liu et al. 2016; Liu et al. 2015). These differences likely reflect sampling methodology more than fundamental differences in the regional arthropod communities themselves. Many desert studies rely primarily on pitfall traps, which, while effective at sampling landscape‐level diversity (Cheli and Corley 2010; Montgomery et al. 2021), are biased toward cursorial taxa and often capture nocturnal ground beetles that avoid daytime heat through nighttime activity (Adis 1979; Niu et al. 2020; Yi et al. 2012). In contrast, our standardized visual surveys recorded arthropods directly on plant surfaces, allowing us to quantify plant occupancy rather than incidental movement across the desert floor. This distinction is important for interpreting arthropod–plant associations, as plant‐based observations allow inference about resource use and structural habitat use, including interactions consistent with nectar exploitation or thermal refuge, which cannot be directly resolved from ground‐based sampling alone. Furthermore, these daytime surveys emphasize diurnally active taxa, particularly flower visitors, likely contributing to the observed dominance of dipterans in this study.

However, interpretations of our observed arthropod‐plant associations must be considered alongside the temporal and observational constraints of the study design. Because surveys were limited to a two‐day window and daytime observations, nocturnal or cryptic species are likely underrepresented, and total species richness associated with *A. erosa* is likely higher than reported here. This limited sampling period also does not allow us to determine whether the high arthropod abundance and morphospecies richness observed on *A. erosa* are specific to midsummer or reflect broader seasonal patterns. In addition, this study was observational and did not experimentally disentangle structural refuge effects from trophic resource effects. Future research should employ experimental manipulations of plant architecture and floral presence, paired with direct measurements of plant microclimate and surface moisture within and around focal *A. erosa* individuals, to better evaluate the relative contributions of structural refugia versus concentrated trophic resources. Such efforts would further clarify the specific mechanisms by which *A. erosa* functions as both a focal resource patch and a seasonal refugium in xeric environments.

Taken together, our results suggest that *A. erosa* functions not only as a localized resource patch but as a structurally mediated microhabitat that supports arthropods across trophic and behavioral roles. Consistently high arthropod abundance, morphospecies richness, and near‐ubiquitous occupancy on plants across sites indicate that the role of this species extends beyond any single plant structure or use (e.g., nectar‐feeding, refuge, hunting) during periods of pronounced resource limitation in the Great Basin Desert (Caldwell 1985). In xeric systems defined by temporally constrained resources (Noy‐Meir 1973; Whitford and Duval 2019), plant species that maintain both structural habitat and concentrated trophic resources into midsummer may support localized concentrations of arthropod abundance and diversity when such resources are scarce in the surrounding habitat. However, because much of our current understanding of desert arthropods is derived from ground‐based sampling methods such as pitfall trapping, the full extent of these plant‐based associations remains under‐characterized. Baseline natural history studies of this kind are needed to clarify how aboveground plant traits organize arthropod communities (Franklin et al. 2016; Lortie et al. 2016). As climate change continues to increase the frequency and intensity of extreme heat in arid environments (Cook et al. 2015; Intergovernmental Panel On Climate Change 2023), plant species that provide both foraging resources and thermally buffered refugia are likely to play a disproportionate role in structuring arthropod communities. Characterizing these focal resources is therefore essential for predicting the future persistence of desert arthropod communities and the continued delivery of the ecosystem services they provide (Tews et al. 2004; Whitford 2000).

## Author Contributions

**Aramee C. Diethelm:** conceptualization (equal), data curation (supporting), formal analysis (lead), investigation (equal), methodology (equal), project administration (lead), supervision (lead), validation (supporting), visualization (equal), writing – original draft (equal), writing – review and editing (equal). **Haylie R. Wilcox:** conceptualization (equal), investigation (equal), methodology (equal), visualization (equal), writing – original draft (equal), writing – review and editing (equal). **Herron N. Renegado:** conceptualization (supporting), data curation (lead), formal analysis (supporting), investigation (equal), methodology (equal), validation (lead), visualization (lead), writing – original draft (equal), writing – review and editing (equal).

## Funding

The authors have nothing to report.

## Conflicts of Interest

The authors declare no conflicts of interest.

## Data Availability

The data that support the findings of this study will be openly available in Dryad at https://doi.org/10.5061/dryad.2v6wwq03z.
